# Reduced microglia activity in patients with long-term immunosuppressive therapy after liver transplantation

**DOI:** 10.1007/s00259-021-05398-w

**Published:** 2021-05-12

**Authors:** Meike Dirks, Ralph Buchert, Ann-Katrin Wirries, Henning Pflugrad, Gerrit M. Grosse, Carlotta Petrusch, Christian Schütze, Florian Wilke, Martin Mamach, Linda Hamann, Laura B. N. Langer, Xiao-Qi Ding, Hannelore Barg-Hock, Jürgen Klempnauer, Christian H. Wetzel, Mario Lukacevic, Eike Janssen, Mariella Kessler, Frank M. Bengel, Lilli Geworski, Rainer Rupprecht, Tobias L. Ross, Georg Berding, Karin Weissenborn

**Affiliations:** 1 Department of Neurology, Hannover Medical School, Carl-Neuberg-Str. 1, 30625 Hannover, Germany; 2 Integrated Research and Treatment Centre Transplantation (IFB-Tx), Hannover Medical School, Hannover, Germany; 3 Department of Diagnostic and Interventional Radiology and Nuclear Medicine, University Medical Center Hamburg-Eppendorf, Hamburg, Germany; 4 Department of Medical Physics and Radiation Protection, Hannover Medical School, Hannover, Germany; 5 Department of Nuclear Medicine, Hannover Medical School, Hannover, Germany; 6 Institute of Diagnostic and Interventional Neuroradiology, Hannover Medical School, Hannover, Germany; 7 General, Visceral and Transplant Surgery, Hannover Medical School, Hannover, Germany; 8 Department of Psychiatry and Psychotherapy, University of Regensburg, Regensburg, Germany

**Keywords:** Cognitive function, ^18^F-GE-180, Imaging, Immunosuppression, Translocator protein (TSPO)

## Abstract

**Purpose:**

Calcineurin inhibitors (CNI) can cause long-term impairment of brain function. Possible pathomechanisms include alterations of the cerebral immune system. This study used positron emission tomography (PET) imaging with the translocator protein (TSPO) ligand ^18^F-GE-180 to evaluate microglial activation in liver-transplanted patients under different regimens of immunosuppression.

**Methods:**

PET was performed in 22 liver-transplanted patients (3 CNI free, 9 with low-dose CNI, 10 with standard-dose CNI immunosuppression) and 9 healthy controls. The total distribution volume (V_T_) estimated in 12 volumes-of-interest was analyzed regarding TSPO genotype, CNI therapy, and cognitive performance.

**Results:**

In controls, V_T_ was about 80% higher in high affinity binders (*n* = 5) compared to mixed affinity binders (*n* = 3). Mean V_T_ corrected for TSPO genotype was significantly lower in patients compared to controls, especially in patients in whom CNI dose had been reduced because of nephrotoxic side effect.

**Conclusion:**

Our results provide evidence of chronic suppression of microglial activity in liver-transplanted patients under CNI therapy especially in patients with high sensitivity to CNI toxicity.

**Supplementary Information:**

The online version contains supplementary material available at 10.1007/s00259-021-05398-w.

## Introduction

Immunosuppression therapy after liver transplantation (LT) usually consists of calcineurin inhibitors (CNI) combined with mycophenolic acid (MPA) and/or steroids [1]. CNI can induce long-term side effects such as renal dysfunction, malignancy, and cardiovascular diseases [2]. Neurological side effects occur as transient dysfunction [3, 4] as well as cognitive impairment and brain atrophy in the long-term [5]*.* Possible pathophysiological mechanisms are CNI-induced atherosclerosis and microangiopathy causing structural brain alterations [2], CNI-induced mitochondrial dysfunction causing alterations of brain energy metabolism, and alterations of the cerebral immune system that may result in neurodegeneration [6, 7].

The present study used PET with the third-generation ligand (S)-N,N-diethyl-9-(2-^18^F-fluoroethyl)-5-methoxy-2,3,4,9-tetrahydro-1H-carbazole-4-carboxamide (^18^F-GE-180) [8, 9] for the translocator protein (TSPO) to test the hypothesis that long-term immunosuppression with CNI in liver-transplanted patients is associated with reduced TSPO availability in the brain, indicating CNI-associated suppression of microglial activity, which in turn is associated with cognitive impairment.

Microglia cells are the primary immune cells of the central nervous system [10]. On activation, microglia cells upregulate TSPO expression [11]. Microglia cells play important roles not only in the survey of the brain parenchyma for danger-associated patterns and the neuroimmune system’s response to these patterns, but also in physiological conditions related to cognition such as shaping neural circuit activity [12]. Association between cognitive impairment and reduced TSPO expression has been reported in Alzheimer’s disease [13], early-stage schizophrenia [14], alcohol dependence [15], and Hepatitis C–associated encephalopathy [16].

## Materials and methods

### Study subjects

This study is a sub-study of a collaborative research project on the impact of long-term CNI therapy on brain function in patients after liver transplantation funded by the German Federal Ministry of Education and Research (reference number: 01EO1302). In the first part of the overall study, patients (*n* = 85) and healthy controls (*n* = 33) underwent neuropsychological testing and magnetic resonance imaging (MRI) [17], as well as analysis of brain- and T-cell-derived cytokines in blood plasma [18]. Details of the recruitment and eligibility criteria have been described previously and are given in the online supplementary (see subsection “Recruitment and eligibility criteria”) [17]. Additional exclusion criteria for the PET sub-study were pregnancy and contraindication for an arterial cannula in the radial artery.

A subsample of 22 patients and three controls agreed to take part in the PET sub-study. Six additional healthy subjects were recruited specifically for the PET sub-study. Demographics are given in Table 1.

**Table 1:** Demographic and clinical characteristics for patient groups and controls

Subgroups	TSPO-GT	N	Sex (m/f)	Age (years) mean and SD	Education (years) mean and SD	Years since LT mean and SD	Years on standard-dose CNI mean and SD	RBANS Total scale mean and SD
(1) CNI free	All	3	3/0	64.3 ± 9.9	12.0 ± 1.7	13.7 ± 2.1	3.7 ± 3.8	97.0 ± 14.8
	LAB	1	1/0	71	13	13	1	107
	MAB	1	1/0	53	13	12	2	104
	HAB	1	1/0	69	10	16	8	80
(2) CNI low dose	All	9	7/2	60.3 ± 11.4	10.1 ± 1.5	14.0 ± 2.3	3.9 ± 2.1	94.9 ± 14.9
	LAB	–	–	–	–	–	–	–
	MAB	2	2/0	52.5 ± 21.9	9 ± 0.0	14.5 ± 3.5	3.0 ± 1.4	88.0 ± 21.2
	HAB	7	5/2	62.6 ± 8.1	10.4 ± 1.6	13.9 ± 2.3	4.1 ± 2.3	96.9 ± 14.2
(3) CNI standard dose	All	10	7/3	57.1 ± 7.6	10.5 ± 1.6	15.0 ± 6.4	12.8 ± 6.1	97.8 ± 9.5
	LAB	2	2/0	63.0 ± 9.9	9.5 ± 0.7	12.0 ± 5.7	8.5 ± 2.1	87.0 ± 5.6
	MAB	5	4/1	56.8 ± 4.7	10.0 ± 1.2	15.0 ± 6.3	14.4 ± 6.3	101 ± 9.1
	HAB	3	1/ 2	53.7 ± 10.6	12.0 ± 1.7	17.0 ± 8.7	13.0 ± 7.9	99.7 ± 8.7
(4) Controls	All	9	5/4	57.9 ± 9.2	11.6 ± 1.5	–	–	101 ± 9.8
	LAB	1	1/0	63.0	10	–	–	101
	MAB	3	1/ 2	50.0 ± 3.6	12.7 ± 0.6	–	–	105 ± 9.5
	HAB	5	3/2	61.6 ± 9.8	11.2 ± 1.6	–	–	98.6 ± 11.4
	p	0.432	0.480	0.651	0.138	0.832	**0.001** *	0.752

Data are mean ± standard deviation. Statistical analysis with chi-square test (for sex and TSPO genotype) and ANOVA (for age, education, years since LT, years on standard-dose CNI, RBANS Total scale) for the comparison of all 4 groups. If significant (*p*-value in bold), bilateral comparison as post hoc test was performed (see*)

*Subgroup 1 vs 2: *p* = 0.899, 1 vs 3: ***p*** **= 0.035,** 2 vs 3: ***p*** **= 0.001**

*GT*, genotype; *N*, number; *LT*, liver transplantation; *SD*, standard deviation; *CNI*, calcineurininhibitors, *RBANS*, Repeatable Battery for the Assessment of Neuropsychological Status

Nine of the 22 liver-transplanted patients were on low-dose CNI therapy (stable tacrolimus trough plasma levels below 5 μg/L or stable cyclosporine A (CsA) trough levels below 50 μg/L) and ten patients on standard-dose CNI therapy (stable tacrolimus trough levels above 5 μg/L or stable CsA trough levels above 50 μg/L). Only three CNI-free patients had agreed to participate in the PET sub-study. The remaining CNI-free patients included in the main study did not fulfil the eligibility criteria (*n* = 9, most often due to deterioration of their medical condition) or declined participation in the PET sub-study (*n* = 8).

The CNI-free patients had been treated for 3.7 (±3.8) years with CNI after LT and were CNI free for 10.0 (± 2.0) years at the time of the study. The main reason for the reduction or termination of CNI had been CNI-induced kidney toxicity. The immunosuppressive therapy regimen of the patients and the etiology of liver disease are given in the Online Supplementary (Table S1). All patients had normal liver function. Kidney function measured as glomerular filtration rate was mildly decreased in all patient groups (CNI free 66.3 ± 9.7 mL/min, CNI low dose 85.7 ± 26.9 mL/min, CNI standard dose 88.2 ± 28.2 mL/min, *p* = 0.454).

CNI trough level at the time of PET was determined in each patient. Additionally, total CNI dosage (ingested dose over time) for each patient was calculated with last observations carried forward between each measuring point between LT and the study examination date (details in [17]).

### TSPO genotyping

For TSPO polymorphism genotyping, genomic DNA was extracted from whole blood and exon 4 of TSPO gene as well as exon/intron junctions were PCR amplified and sequenced using specific primers (Department of Psychiatry and Psychotherapy, University of Regensburg, Germany, Prof. Wetzel). Sequencing data were analyzed using SnapGene software (GSL Biotech; available at snapgene.com). Subjects were categorized as low (LAB), mixed (MAB), or high (HAB) affinity binders depending on whether none, one, or two copies of the high affinity binding site were present [19–22].

### Magnetic resonance imaging

High-resolution T1-weighted magnetization prepared rapid acquisition gradient echo MRI of the brain for stereotactical normalization of the PET images was acquired from all subjects at 3T (Verio, Siemens, Erlangen, Germany; voxel size 1.0 × 1.0 × 1.0 mm^3^, echo time 2.93 ms, repetition time 1900 ms, inversion time 900 ms, flip angle 9.0°) [19].

### Synthesis of ^18^F-GE-180 and PET/CT imaging

^18^F-GE-180 was produced in a GMP-conform synthesis using a single-use disposable cassette (FASTlab PET GE-180 cassette, GE Healthcare, UK) on an automated synthesis module (FASTlab™, GE Healthcare, UK) and the corresponding S-enantiomeric pure mesylate precursor (3.5 mg, GMP grade) [23]. ^18^F-fluoride was obtained from a 11-MeV cyclotron (Eclipse HP, Siemens, Knoxville, USA) using the ^18^O(p,n)^18^F nuclear reaction on enriched (97–98%) ^18^O-water or obtained from external sources [19].

PET imaging was performed with a Biograph mCT-128 (Siemens, Erlangen, Germany). A low-dose computerized tomography (CT) scan (100 kV, 30 mAs) was acquired for attenuation correction of the PET data. Thereafter, a list mode emission scan of 90-min duration was started simultaneously with intravenous injection of 178 ± 7 MBq (range 157–195) ^18^F-GE-180 over 10s into an antecubital vein. Administered dose of ^18^F-GE-180 was 2.14 ± 0.35 MBq/kg bodyweight (range 1.62–3.11 MBq/kg). Specific dose did not differ between TSPO genotypes (2.13 ± 0.13 MBq/kg, 2.05 ± 0.38 MBq/kg, and 2.19 ± 0.38 MBq/kg in LAB, MAB, and HAB, respectively, analysis of variance (ANOVA) *p* = 0.616). In order to measure the input function for tracer kinetic modeling, arterial blood was drawn from the radial artery. Automatic blood sampling was done during the first 15 min of the PET scan (Veenstra PBS-101, Veenstra Instruments, The Netherlands), followed by manual blood sampling until the end of the PET acquisition. Reconstruction and preprocessing of the PET data have been described previously [19].

### Quantitative analysis of TSPO expression

The total distribution volume (V_T_) of ^18^F-GE-180 was used as the primary quantitative measure of regional TSPO expression in the brain. V_T_ is the sum of the specific distribution volume (V_S_) for specific binding of ^18^F-GE-180 to TSPO plus the non-displaceable distribution volume (V_ND_) comprising free (unbound) and unspecifically bound (not to TSPO) ^18^F-GE-180 in tissue (V_T_ = V_S_ + V_ND_)*.* V_S_ is proportional to the density of TSPO available for binding of ^18^F-GE-180. V_ND_ is background signal of no primary interest. For ^18^F-GE-180, V_S_ is assumed to be larger than V_ND_ in HAB and MAB subjects [22], making V_T_ a useful marker of TSPO availability.

Tissue time activity curves were obtained for the following volumes-of-interest (VOI) using standard VOI masks [19]: frontal, parietal, lateral temporal, occipital, cingulate, and mesial temporal cortex, cerebellum, insula, precuneus, striatum, thalamus, and white matter (superior longitudinal fasciculus). Tissue time activity curves were corrected for blood in brain tissue assuming a fixed fractional blood volume of 5% [24]*.*

V_T_ was determined separately for each VOI using the time activity curve and the invasive graphical Logan method with metabolite-corrected arterial input function [19]. Mean whole brain V_T_ was estimated by the volume-weighted average of all regional V_T_.

Parametric V_T_ images were obtained for each subject by applying the invasive graphical Logan method on a voxel-by-voxel base.

### Neurological and neuropsychological assessment

All participants underwent a neurological examination and completed the Repeatable Battery for the Assessment of Neuropsychological Status (RBANS). The latter assesses attention, visuospatial/constructional ability, language skill, and immediate and delayed memory. An age-corrected index score was calculated for each of these domains. The total age-corrected scale (RBANS Total Scale) was obtained by summing all index scores [25]. Higher test values suggest better cognitive function.

The study has been approved by the ethics committee of Hannover Medical School (reference no. 6525) and the Federal Office for Radiation Protection (reference no.: Z5 – 22463/2 – 2015-030). All subjects provided written informed consent.

### Statistical analyses

The effect of the TSPO genotype on V_T_ was tested in the control subjects using ANOVA with V_T_ as dependent variable and TSPO genotype (HAB versus MAB) and VOI as fixed factors.

The impact of CNI therapy (independent of the dose) on V_T_ was first tested by comparing the group of low-dose and standard-dose patients combined with the controls using ANOVA with V_T_ as dependent variable and group and TSPO genotype as fixed factors. The ANOVA was performed (i) separately for each VOI, (ii) for whole brain V_T_, and (iii) with VOI (*n* = 12) as additional fixed factor.

The impact of the CNI dose on V_T_ was tested by ANOVA comparing V_T_ between patients under low-dose CNI, patients under standard-dose CNI, and controls. TSPO genotype and ROI were taken into account as additional fixed factors.

To evaluate the impact of the CNI plasma level on V_T_, the CNI trough level at the time of PET was transformed to z-scores, separately for tacrolimus and CsA. The impact of the CNI trough level on V_T_ was tested by ANOVA with V_T_ as dependent variable, TSPO genotype and VOI as fixed factors, and the CNI trough level z-score as covariate including all patients under CNI therapy.

An analogous analysis was performed with the total CNI dosage transformed to z-scores.

A potential association between cognition and TSPO availability was first tested by linear regression of each of the RBANS scores as dependent variable and mean whole brain V_T_ or regional V_T_ in one of the 12 different VOIs as predictors. The regression analyses were restricted to the ten HAB subjects amongst the patients with CNI therapy, because including the TSPO genotype as additional predictor variable in the regression model would have caused multicollinearity issues that limit the interpretation of the regression coefficients of the individual predictor variables.

In order to increase the sample size for testing of potential associations between cognition and TSPO availability by combining HAB and MAB patients with CNI therapy (*n* = 17) in a single analysis, the polymorphism plot was used to estimate the non-displaceable distribution volume (V_ND_) of ^18^F-GE-180 (for further details, see Online Supplementary subsection “Polymorphism plot of ^18^F-GE-180 for the preparation of testing for an association between cognition data and TSPO expression”) [22, 26]. The specific distribution volume (V_S_) was then computed as V_S_ = V_T_–V_ND_. The specific distribution volume of the MAB subjects was multiplied by the factor 2 to account for the additional copy of the high affinity binding site in the HAB patients. A potential association between cognition and TSPO expression was tested by linear regression of the RBANS scores as dependent variable and mean whole brain V_S_ or regional V_S_ in one of the VOIs as predictors.

A two-sided *p*-value <0.05 was considered significant. SPSS Version 25.0 was used for statistical analysis.

## Results

### Characteristics of study subjects

Patients and controls did not significantly differ regarding age, sex, and education (Table 1). RBANS performance was better in controls than in patients, but the difference did not reach statistical significance (ANOVA with 4 subgroups *p* = 0.752, Table 1). The patients performed worse than controls especially regarding immediate memory (*p* = 0.077). Detailed data are given in Table 2.

**Table 2:** Results of the RBANS

Subgroups	N	RBANS Immediate memory	RBANS Visuospatial/constructional	RBANS Language	RBANS Attention	RBANS Delayed memory	RBANS Total scale
(1) CNI free	3	97.7 ± 17.1	88.0 ± 19.3	109.0 ± 10.5	108.0 ± 3.5	86.3 ± 29.8	97.0 ± 14.8
(2) CNI low dose	9	95.9 ± 11.3	89.2 ± 12.7	102.4 ± 14.9	98.7 ± 18.5	91.6 ± 11.9	94.9 ± 14.9
(3) CNI standard dose	10	101.5 ± 15.8	94.1 ± 16.5	97.0 ± 12.2	101.2 ± 17.8	101.4 ± 5.2	97.8 ± 9.5
(4) Controls	9	108.4 ± 12.6	86.8 ± 17.6	104.4 ± 10.3	105.9 ± 20.9	100.0 ± 9.9	101 ± 9.8
P (all groups)		0.282	0.782	0.419	0.797	0.138	0.752
P (P/C)		0.077	0.474	0.476	0.504	0.360	0.332

Values are given in mean ± standard deviation. Statistical analysis was performed with ANOVA (all 4 groups) and unpaired Student’s t-test (patient group vs. control group)

*RBANS*, Repeatable Battery for the Assessment of Neuropsychological Status; *P*, patients; *C*, controls

TSPO genotyping in the nine controls revealed one LAB (11%), three MAB (33%), and five HAB (56%). The patient group (*n* = 22) showed a similar distribution: three LAB (14%), eight MAB (36%), and eleven HAB (50%) (chi-square *p* = 0.958, Table 1).

### TSPO genotype effect on V_T_ in the control subjects

The 5 HAB controls were on average 11.7 years older than the 3 MAB controls (62.2 ± 9.5 versus 50.5 ± 2.3 years, *p* = 0.049).

**Fig. 1:**
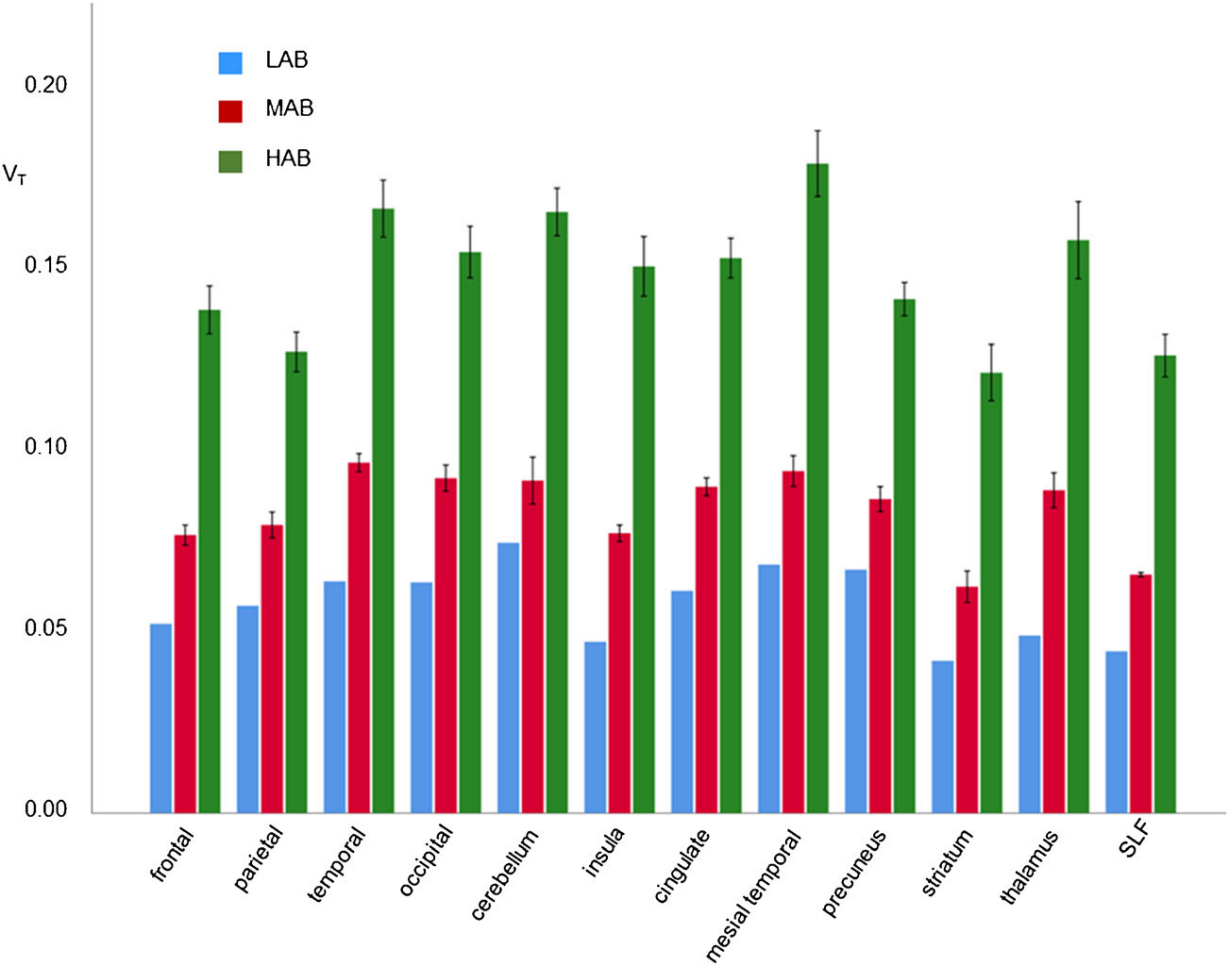
Mean and standard deviation of V_T_ in the control group separated for brain region and TSPO genotype (LAB, MAB, HAB). HAB, high affinity binder *n* = 5; LAB, low affinity binder *n* = 1; MAB, mixed affinity binder *n* = 3; SLF, superior longitudinal fasciculus; TSPO, translocator protein; V_T_, distribution volume

Mean estimated V_T_ over all VOIs was significantly higher in HAB (0.148, 95%-CI: 0.144–0.151) compared to MAB controls (0.083, 0.079–0.088; *p* < 0.0005) (Fig. 1). The TSPO genotype effect on V_T_ did not differ between VOIs (genotype*VOI interaction: *p* = 0.502), although V_T_ differed significantly between VOIs (p < 0.0005). V_T_ was largest in the mesiotemporal lobe and smallest in the striatum (Fig. 1).

### Impact of CNI therapy on V_T_ independent of dose

ANOVA of age with group (CNI therapy versus controls) and TSPO genotype (HAB versus MAB subjects) as fixed factors did not reveal a significant age difference between patients and controls (*p* = 0.671) nor between HAB and MAB subjects (*p* = 0.057).

ANOVA of V_T_ with group (CNI therapy versus controls) and TSPO genotype as fixed factors, performed separately for each VOI and for the whole brain, confirmed higher V_T_ in HAB compared to MAB subjects (*p* ≤ 0.018 in 11 VOIs and in whole brain, *p* = 0.068 in the remaining (parietal) VOI). V_T_ was lower in patients under CNI therapy in 11 of the 12 VOIs and in whole brain, but the level of statistical significance was not reached in any of them (*p* ≥ 0.073).

**Fig. 2:**
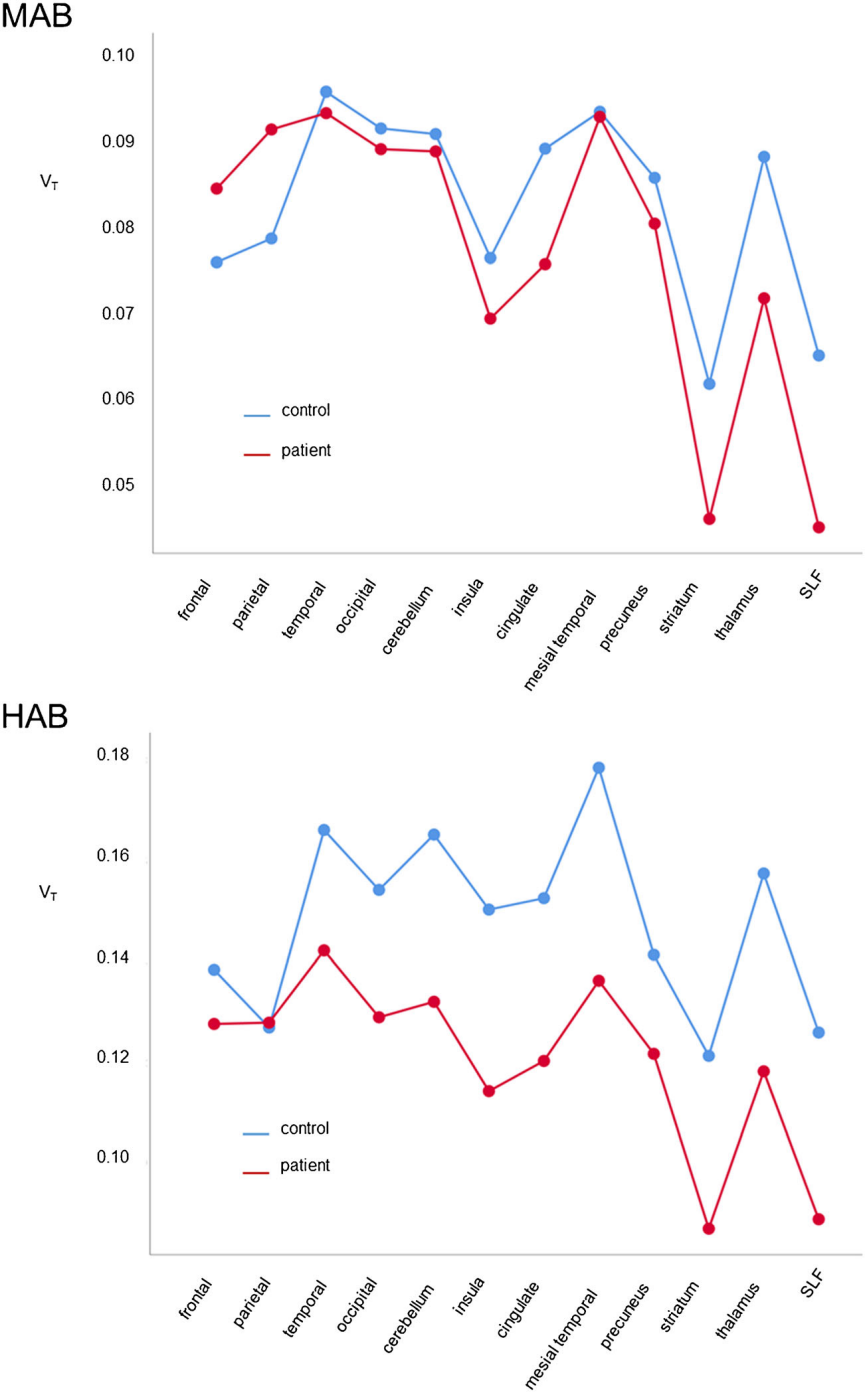
Mean of V_T_ in different brain regions from controls and patients separated according to TSPO genotype (MAB, HAB)/ HAB, high affinity binder; MAB, mixed affinity binder; SLF, superior longitudinal fasciculus; TSPO, translocator protein; V_T_, total distribution volume

ANOVA of V_T_ with group (CNI therapy versus controls), TSPO genotype, and VOI as fixed factors also confirmed higher V_T_ in HAB compared to MAB subjects: estimated mean V_T_ = 0.134 (95%-CI: 0.128–0.140) versus 0.080 (0.073–0.088) (*p* < 0.0005). The TSPO genotype explained 30.3% of the between-subjects variance of V_T_ (η^2^ = 0.303). The VOI dependence of V_T_ was also confirmed (*p* = 0.002, η^2^ = 0.106). The reduction of V_T_ in patients under CNI therapy compared to controls now reached statistical significance (*p* = 0.001, η^2^ = 0.040): estimated mean V_T_ = 0.099 (95%-CI 0.093–0.104) versus 0.115 (0.107–0.124). The reduction of V_T_ in patients under CNI therapy was more pronounced in HAB subjects than in MAB subjects (genotype*group interaction *p* = 0.029, η^2^ = 0.019) (Fig. 2). When ANOVA of V_T_ with group (patients with CNI therapy versus controls) and VOI as fixed factors was performed separately for HAB and MAB subjects, the group effect (lower V_T_ in patients with CNI therapy) was highly significant in the HAB subjects (*p* < 0.0005), but it failed to reach statistical significance in the MAB subjects (*p* = 0.106).

Age did not show a significant effect on V_T_ when it was included as a covariate in the ANOVA of V_T_ with group (CNI therapy versus controls), TSPO genotype, and VOI as fixed factors (*p* = 0.674). All other effects were confirmed (TSPO genotype *p* < 0.0005, group *p* = 0.002, VOI p = 0.002, genotype*group interaction *p* = 0.026).

### Impact of CNI dose regimen on V_T_

**Fig. 3:**
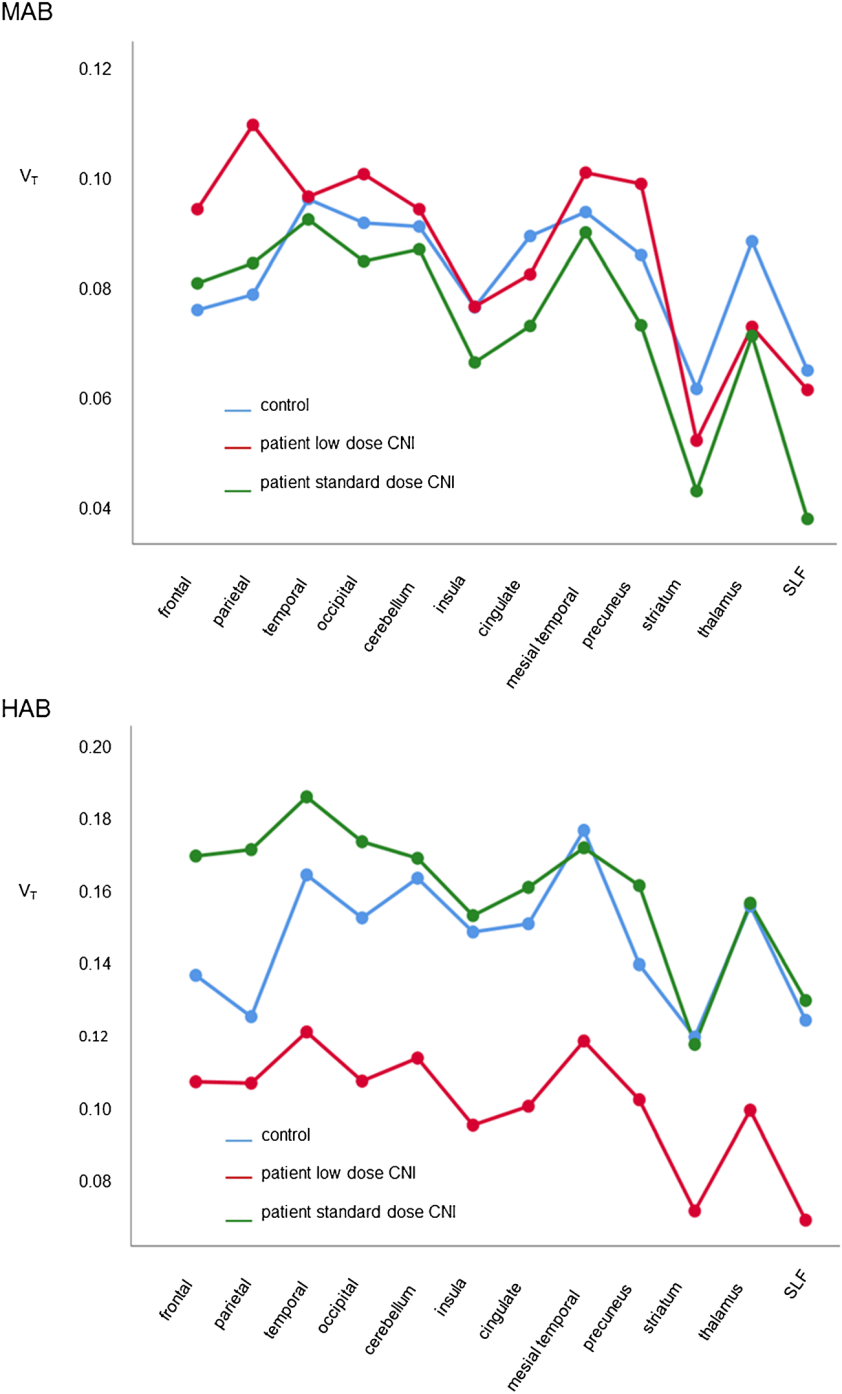
Mean V_T_ in 12 brain regions from MAB and HAB subjects separated according to controls, patients on low-dose CNI and standard-dose CNI. CNI, calcineurininhibitors; HAB, high affinity binder; MAB, mixed affinity binder; SLF, superior longitudinal fasciculus; V_T_, total distribution volume

ANOVA of V_T_ with group (low dose versus standard-dose CNI versus controls), TSPO genotype, and VOI as fixed factors showed a significant group effect (*p* < 0.0005). Post hoc testing using Scheffe’s test showed V_T_ to be significantly lower in patients under low-dose CNI therapy compared to controls (p < 0.0005): estimated mean V_T_ = 0.095 (0.086–0.103) versus 0.115 (0.108–0.123). In patients under standard-dose CNI therapy, estimated mean V_T_ was 0.118 (0.110–0.125), similar to controls. Figure 3 shows V_T_ for each brain region separated according to TSPO genotype and CNI therapy.

ANCOVA with V_T_ as dependent variable, TSPO genotype (HAB versus MAB) and VOI as fixed factors, and the CNI trough level z-score as covariate including all patients under CNI therapy, independent of the specific CNI, revealed a significant effect of the CNI trough level (p < 0.0005) that explained 11.0% of the between-subjects variance of V_T_ (η^2^ = 0.110). The model coefficient of the CNI trough level z-score was positive (B = 0.018, 95%-CI 0.010–0.025) indicating lower V_T_ at lower CNI trough level.

The total CNI dose explained less V_T_ variance (3.1%) compared to CNI trough level.

**Fig. 4:**
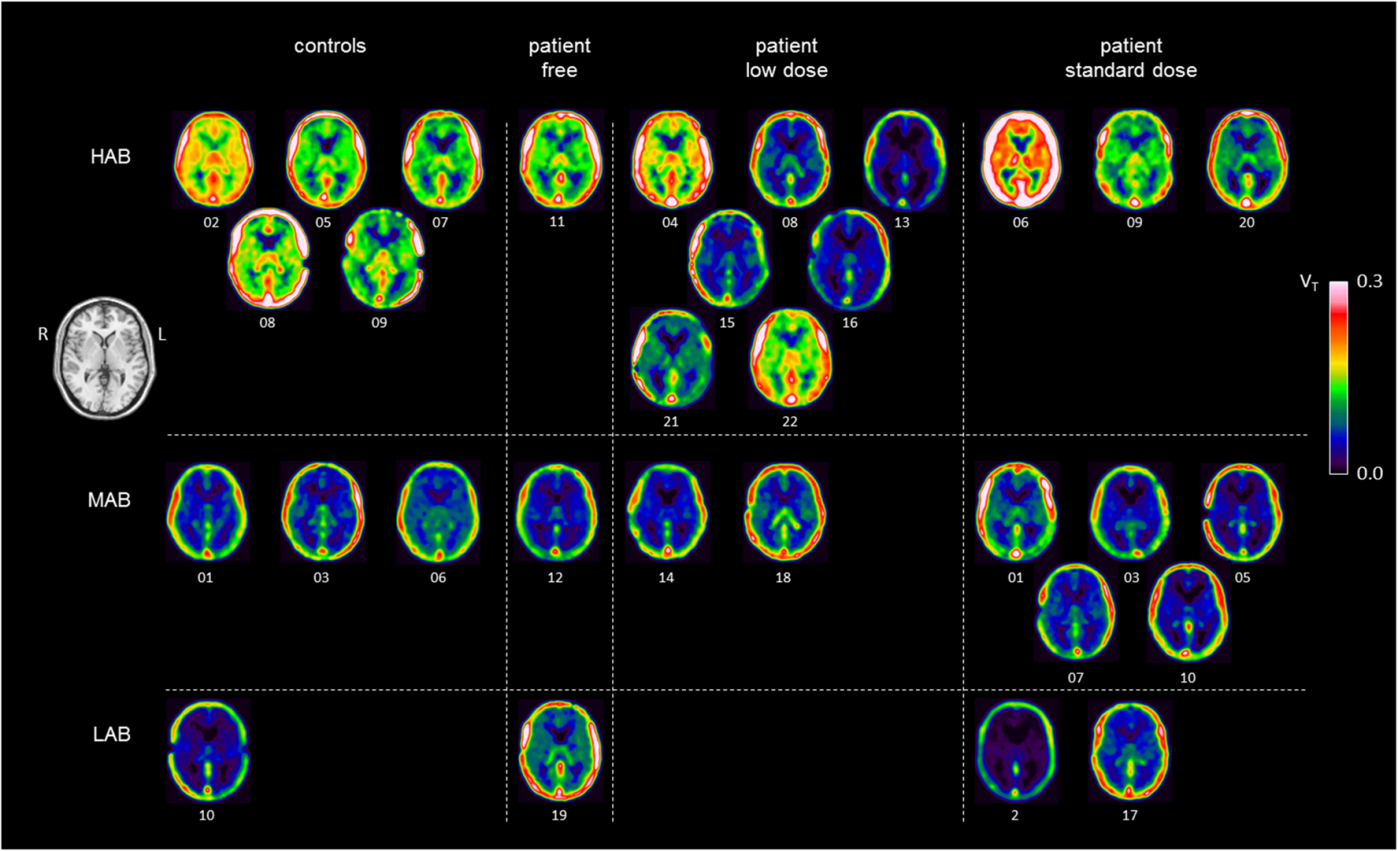
Representative transversal slices of the individual voxel-based V_T_ maps for all study participants separated according to subgroup and TSPO genotype. The figure illustrates the TSPO genotype effect on V_T_ (HAB > MAB > LAB) in general and in particular, e.g., a reduction of V_T_ in the patients compared to controls that is mainly driven by HAB patients under low-dose CNI therapy. CNI, calcineurininhibitors; HAB, high affinity binder; L, left; LAB, low affinity binder; MAB, mixed affinity binder; V_T_, total distribution volume; R, right

Figure 4 displays a representative transversal slice of the individual voxel-based V_T_ maps for all study participants separated according to group and TSPO genotype. The figure illustrates the TSPO genotype effect on V_T_ (HAB > MAB > LAB) as well as a reduction of V_T_ in the patients compared to controls that is mainly driven by HAB patients under low-dose CNI therapy.

### Association of cognition with TSPO availability

None of the 78 (= 6*13) linear regression analyses of the five RBANS index scores or the RBANS total scale with one of the 13 V_T_ or Vs values (whole brain and 12 regional VOIs) as predictor in the 10 or 17 HAB patients with low- or standard-dose CNI therapy achieved statistical significance.

## Discussion

The main finding of this study was the reduced cerebral TSPO availability in liver-transplanted patients under CNI therapy, the standard immunosuppressive therapy after liver transplantation [1]. This finding supports the primary hypothesis of the study that CNI therapy causes chronic suppression of microglial activity in liver-transplanted patients. Thus, CNI not only play a role as suppressors of peripheral cell-mediated immune reactions, but they are also involved in the regulation of the cerebral immune system.

So far this hypothesis was mainly based on experimental research. Tacrolimus and, to lesser extent, CsA inhibit microglia activation in cultured cells [27]. Tacrolimus reduced LPS-induced activation of mitogen-activated protein kinase (MAPK) and NFκB signaling pathways, crucial steps in microglia activation. This led to an inhibition of morphological transformation, motility, and release of mediators like IL-1β, Cox-2, and iNOS in inflammatory microglia [27]. Tacrolimus also attenuated microgliosis in a mouse model of Alzheimer’s disease [28].

Interestingly, the reduction of TSPO availability under CNI therapy was mainly driven by the patients under low-dose therapy, whereas the patients under standard dose showed similar TSPO availability as controls. This finding was not unexpected, as it is consistent with the results of a previous sub-study of our collaborative research demonstrating more prominent reduction of cognitive performance in liver-transplanted patients who initially had shown nephrotoxic side effects of standard-dose CNI therapy and therefore were on low-dose CNI therapy compared to liver-transplanted patients who had not shown relevant CNI nephrotoxicity and therefore remained on standard-dose CNI therapy (Figure 4 in [17]). Liver-transplanted patients on low-dose CNI therapy also showed more microvascular pathology (white matter hyperintensities in MRI) in the occipital lobe than liver-transplanted patients with standard-dose CNI therapy (Figure 5 in [17]), although the difference did not reach statistical significance. Of note, at the time point of the PET study, both patient groups with CNI therapy showed similar glomerular filtration rates. In addition, only 2 of 9 (22%) of the low-dose and 1 of 10 (10%) of the standard-dose CNI therapy group showed chronic kidney disease grade III. Our hypothesis suggests that nephrotoxic CNI side effects might be an indicator of generally increased susceptibility against unwanted CNI effects including side effects on the cerebral immune system. The results of the present study provide further evidence in favor of this hypothesis.

Current CNI trough levels explained more variability of TSPO availability than the CNI total dose. This finding might favor an acute (possibly reversible) effect of the CNI therapy on microglial activity rather than a cumulative long-term effect.

This study demonstrated ^18^F-GE-180 V_T_ to be considerably higher in HAB subjects compared to MAB subjects (Fig. 1), similar to other 2nd and 3rd generation TSPO ligands for PET [29]. This is an important finding, because previous studies with ^18^F-GE-180 PET reported inconsistent results. Feeney and co-workers found no significant effect of the TSPO gene polymorphism on any regional V_T_ in a study with dynamic ^18^F-GE-180 PET in 10 healthy subjects (5 MAB and 5 HAB) [24], neither did Unterrainer and co-workers, performing static ^18^F-GE-180 PET in 19 patients with multiple sclerosis (3 LAB, 5 MAB, 11 HAB) [30]. In contrast, Fan and co-workers, using dynamic ^18^F-GE-180 PET in 10 healthy subjects (6 HAB, 4 MAB), found regional V_T_ to be higher in HAB than in MAB subjects [20]. A study in patients with multiple sclerosis (3 HAB, 3 MAB) found whole brain V_T_ to be about 70% larger in HAB compared to MAB subjects [22]. The present study adds further evidence of a strong effect of the TSPO genotype on ^18^F-GE-180 binding. As a consequence, TSPO genotype was taken into account in all analyses. We recommend that future studies with ^18^F-GE-180 PET also account for TSPO genotype.

Reduction of the total distribution volume (V_T_) of ^18^F-GE-180 under CNI therapy was mainly driven by HAB subjects. Given that specific binding of ^18^F-GE-180 to TSPO accounts for a larger proportion of V_T_ in HAB subjects than in MAB subjects, this finding suggests that CNI therapy mainly affects specific binding (via suppression of microglial activation) whereas non-displaceable background signal is not affected by CNI therapy. This supports the use of ^18^F-GE-180 PET to detect CNI-induced alterations of microglia status. Furthermore, higher effect size of CNI-induced V_T_ alterations in HAB subjects compared to MAB subjects provides higher power of ^18^F-GE-180 PET to detect and monitor CNI-induced microglial alterations. Future studies with ^18^F-GE-180 PET might restrict enrolment to HAB subjects in order to reduce the sample size required to provide the statistical power to detect a given effect size.

The spatial distribution pattern of ^18^F-GE-180 V_T_ in the HAB control subjects (see Online Supplementary Figure S2) was in reasonable agreement with the distribution of microglia in the healthy brain shown in preclinical immunocytochemical studies [31]: ^18^F-GE-180 V_T_ was higher in gray than in white matter, and it was particularly high in the mesial temporal cortex and in the thalamus. Differences between the spatial distribution pattern of ^18^F-GE-180 V_T_ and the microglia density measured by immunocytochemical methods to some extent might be explained by the fact that TSPO expression in the brain is not restricted to microglia. TSPO is expressed in ependymal and vascular endothelial cells, but also in most other cells including neurons, although at lower levels [32]. As a consequence, TSPO not associated to microglia contributes to specific binding of ^18^F-GE-180. Partial volume effects caused by limited spatial resolution of PET also contribute to the differences between the spatial distribution pattern of ^18^F-GE-180 V_T_ and the microglia density measured by immunocytochemical methods. Finally, variability of ^18^F-GE-180 V_T_ between brain regions is expected to underestimate variability of microglia density, because only the specific distribution volume V_S_ of ^18^F-GE-180 is assumed to be proportional to TSPO density. The non-displaceable distribution volume V_ND_ dilutes spatial variability of the specific contribution in V_T_ (V_T_ = V_S_ + V_ND_).

The following limitations of this study should be noted. First, the sample size was rather small given that different CNI dose regimes and different TSPO genotypes were included. This resulted in low statistical power to detect reduced cognitive performance in liver-transplanted patients on CNI therapy described in previous studies as well as its possible association with reduced global/regional TSPO availability in the brain. Furthermore, the group of CNI-free patients included only three subjects, one of each TSPO genotype (Table 1), which precluded statistical comparison of the CNI-free patients with the other subgroups.

Second, the total distribution volume V_T_ of ^18^F-GE-180 was in general very low throughout the whole brain. In the HAB controls, regional V_T_ ranged between 0.12 and 0.18 mL/cm^3^ (Fig. 1). This is in line with previous ^18^F-GE-180 studies in healthy human subjects [20, 22, 24, 33] and most likely is due to a low permeability-surface-area-product of brain capillaries for ^18^F-GE-180 as suggested by the small rate constant K_1_ for unidirectional transport of ^18^F-GE-180 from arterial blood to tissue even at normal cerebral blood flow [20, 24]. For a systematic comparison of 13 different TSPO tracers including [^11^C](R)-PK11195 and ^18^F-GE-180, we refer the reader to the excellent review by Cumming and co-workers [29]. However, Sridharan and co-workers recently confirmed specific binding of ^18^F-GE-180 in the human brain by a blocking study in multiple sclerosis patients [22]. These authors found that in HAB subjects about 57% of V_T_ represent specific binding of ^18^F-GE-180 to the TSPO [22]. This high specific-to-non-displaceable binding ratio makes ^18^F-GE-180 a useful tracer for the detection and quantitative characterization of altered TSPO availability in the human brain, particularly in HAB subjects, despite its low extraction from arterial blood across the intact blood-brain barrier.

Third, ^18^F-GE-180 V_T_ maps generated by voxel-based tracer kinetic modeling demonstrated considerable between-subjects variability also within subgroups separated according to TSPO genotype and clinical group (Fig. 4). In particular, three of the ten HAB patients under low- or standard-dose CNI therapy (patients 4, 6, 22) presented with higher V_T_ compared to the other seven patients with CNI therapy (Fig. 4). A possible explanation of this finding is a gender effect on V_T_, as these three patients were all females, compared to only one of the remaining seven HAB patients (patient 9). A recent study reported about 25% higher whole gray matter V_T_ of the TSPO ligand [^11^C]PBR28 in healthy HAB females compared to healthy HAB males [34]. However, amongst the five HAB controls included in the present study, the two females (controls 2, 7) did not differ from the three males with respect to V_T_ (Fig. 4). Thus, the present study does not provide clear evidence of a gender effect on ^18^F-GE-180 V_T._ Nevertheless, a gender effect cannot be ruled out and, therefore, might have contributed to the reduction of V_T_ in the liver-transplanted patients on CNI therapy (HAB female to HAB male ratio was 5:2 in the low-dose CNI patients compared to 3:2 in the control subjects, Table 1). Future studies should balance subsamples with respect to gender.

Finally, it cannot be ruled out that slightly reduced RBANS performance was pre-existing in the patients included in the present cross-sectional study (prior to liver transplantation and CNI therapy). The RBANS is sensitive to cognitive changes caused by medical treatments, but it also depends on premorbid intellectual functioning [35].

In conclusion, the findings of this study provide evidence of chronic suppression of microglial activity in liver-transplanted patients under CNI therapy with reduced dose due to high sensitivity to CNI toxicity as indicated by nephrotoxicity at the standard dose. This finding might have considerable impact on the management of liver-transplanted patients, given that microglia plays important roles in the survey of the brain parenchyma for danger-associated patterns and the neuroimmune system’s response to these patterns as well as in physiological conditions whose disturbance might result in psychiatric and neurological symptoms.

## Supplementary Information

ESM 1 (DOCX 1.82 mb).

## References

[1] Herzer K, Strassburg CP, Braun F, Engelmann C, Guba M, Lehner F (2016). Selection and use of immunosuppressive therapies after liver transplantation: current German practice. Clin Transpl.

[2] Johnston SD, Morris JK, Cramb R, Gunson BK, Neuberger J (2002). Cardiovascular morbidity and mortality after orthotopic liver transplantation. Transplantation..

[3] Bernhardt M, Pflugrad H, Goldbecker A, Barg-Hock H, Knitsch W, Klempnauer J (2015). Central nervous system complications after liver transplantation: common but mostly transient phenomena. Liver Transpl.

[4] Rompianesi G, Montalti R, Cautero N, De Ruvo N, Stafford A, Bronzoni C (2015). Neurological complications after liver transplantation as a consequence of immunosuppression: univariate and multivariate analysis of risk factors. Transpl Int.

[5] Senzolo M, Pizzolato G, Ferronato C, Chierichetti F, Boccagni P, Dam M (2009). Long-term evaluation of cognitive function and cerebral metabolism in liver transplanted patients. Transplant Proc.

[6] Chen CC, Hsu LW, Huang LT, Huang TL (2010). Chronic administration of cyclosporine A changes expression of BDNF and TrkB in rat hippocampus and midbrain. Neurochem Res.

[7] Illsinger S, Janzen N, Lucke T, Bednarczyk J, Schmidt KH, Hoy L (2011). Cyclosporine A: impact on mitochondrial function in endothelial cells. Clin Transpl.

[8] Dickens AM, Vainio S, Marjamaki P, Johansson J, Lehtiniemi P, Rokka J (2014). Detection of microglial activation in an acute model of neuroinflammation using PET and radiotracers 11C-(R)-PK11195 and 18F-GE-180. J Nucl Med.

[9] Wadsworth H, Jones PA, Chau WF, Durrant C, Fouladi N, Passmore J (2012). [(1)(8)F]GE-180: a novel fluorine-18 labelled PET tracer for imaging Translocator protein 18 kDa (TSPO). Bioorg Med Chem Lett.

[10] Heneka MT, Kummer MP, Latz E (2014). Innate immune activation in neurodegenerative disease. Nat Rev Immunol.

[11] Chen MK, Guilarte TR (2008). Translocator protein 18 kDa (TSPO): molecular sensor of brain injury and repair. Pharmacol Ther.

[12] Wake H, Moorhouse AJ, Nabekura J (2011). Functions of microglia in the central nervous system--beyond the immune response. Neuron Glia Biol.

[13] Xu JB, Sun JJ, Perrin RJ, Mach RH, Bales KR, Morris JC (2019). Translocator protein in late stage Alzheimer’s disease and dementia with Lewy bodies brains. Ann Clin Transl Neurol.

[14] Collste K, Plaven-Sigray P, Fatouros-Bergman H, Victorsson P, Schain M, Forsberg A (2017). Lower levels of the glial cell marker TSPO in drug-naive first-episode psychosis patients as measured using PET and [C-11]PBR28. Mol Psychiatry.

[15] Kalk NJ, Guo Q, Owen D, Cherian R, Erritzoe D, Gilmour A (2017). Decreased hippocampal translocator protein (18kDa) expression in alcohol dependence: a [C-11] PBR28 PET study. Transl Psychiatry.

[16] Pflugrad H, Meyer GJ, Dirks M, Raab P, Tryc AB, Goldbecker A (2016). Cerebral microglia activation in hepatitis C virus infection correlates to cognitive dysfunction. J Viral Hepat.

[17] Pflugrad H, Schrader AK, Tryc AB, Ding X, Lanfermann H, Jackel E (2018). Longterm calcineurin inhibitor therapy and brain function in patients after liver transplantation. Liver Transpl.

[18] Dirks M, Pflugrad H, Tryc AB, Schrader AK, Ding X, Lanfermann H (2020). Impact of immunosuppressive therapy on brain derived cytokines after liver transplantation. Transpl Immunol.

[19] Buchert R, Dirks M, Schutze C, Wilke F, Mamach M, Wirries AK (2020). Reliable quantification of (18)F-GE-180 PET neuroinflammation studies using an individually scaled population-based input function or late tissue-to-blood ratio. Eur J Nucl Med Mol Imaging.

[20] Fan Z, Calsolaro V, Atkinson RA, Femminella GD, Waldman A, Buckley C (2016). Flutriciclamide (F-18-GE180) PET: first-in-human PET study of novel third-generation in vivo marker of human translocator protein. J Nucl Med.

[21] Owen DR, Yeo AJ, Gunn RN, Song K, Wadsworth G, Lewis A (2012). An 18-kDa translocator protein (TSPO) polymorphism explains differences in binding affinity of the PET radioligand PBR28. J Cereb Blood Flow Metab.

[22] Sridharan S, Raffel J, Nandoskar A, Record C, Brooks DJ, Owen D (2019). Confirmation of specific binding of the 18-kDa translocator protein (TSPO) radioligand [(18)F]GE-180: a blocking study using XBD173 in multiple sclerosis Normal appearing white and grey matter. Mol Imaging Biol.

[23] Wickstrom T, Clarke A, Gausemel I, Horn E, Jorgensen K, Khan I (2014). The development of an automated and GMP compliant FASTlab synthesis of [(18) F]GE-180; a radiotracer for imaging translocator protein (TSPO). J Label Compd Radiopharm.

[24] Feeney C, Scott G, Raffel J, Roberts S, Coello C, Jolly A (2016). Kinetic analysis of the translocator protein positron emission tomography ligand [F-18]GE-180 in the human brain. Eur J Nucl Med Mol Imaging.

[25] Randolph C (1998). Repeatable battery for the assessment of neuropsychological status (RBANS).

[26] Guo Q, Colasanti A, Owen DR, Onega M, Kamalakaran A, Bennacef I (2013). Quantification of the specific translocator protein signal of 18F-PBR111 in healthy humans: a genetic polymorphism effect on in vivo binding. J Nucl Med.

[27] Zawadzka M, Dabrowski M, Gozdz A, Szadujkis B, Sliwa M, Lipko M (2012). Early steps of microglial activation are directly affected by neuroprotectant FK506 in both in vitro inflammation and in rat model of stroke. J Mol Med (Berl).

[28] Rojanathammanee L, Floden AM, Manocha GD, Combs CK (2015). Attenuation of microglial activation in a mouse model of Alzheimer’s disease via NFAT inhibition. J Neuroinflammation.

[29] Cumming P, Burgher B, Patkar O, Breakspear M, Vasdev N, Thomas P (2018). Sifting through the surfeit of neuroinflammation tracers. J Cereb Blood Flow Metab.

[30] Unterrainer M, Mahler C, Vomacka L, Lindner S, Havla J, Brendel M (2018). TSPO PET with [(18)F]GE-180 sensitively detects focal neuroinflammation in patients with relapsing-remitting multiple sclerosis. Eur J Nucl Med Mol Imaging.

[31] Lawson LJ, Perry VH, Dri P, Gordon S (1990). Heterogeneity in the distribution and morphology of microglia in the normal adult mouse brain. Neuroscience..

[32] Notter T, Schalbetter SM, Clifton NE, Mattei D, Richetto J, Thomas K, et al. Neuronal activity increases translocator protein (TSPO) levels. Mol Psychiatry. 2020. 10.1038/s41380-020-0745-1. 10.1038/s41380-020-0745-1 PMC8440208 32398717

[33] Zanotti-Fregonara P, Pascual B, Rizzo G, Yu M, Pal N, Beers D (2018). Head-to-head comparison of (11)C-PBR28 and (18)F-GE180 for quantification of the translocator protein in the human brain. J Nucl Med.

[34] Tuisku J, Plaven-Sigray P, Gaiser EC, Airas L, Al-Abdulrasul H, Bruck A (2019). Effects of age, BMI and sex on the glial cell marker TSPO - a multicentre [(11)C]PBR28 HRRT PET study. Eur J Nucl Med Mol Imaging.

[35] Duff K, Dalley BCA, Suhrie KR, Hammers DB (2019). Predicting premorbid scores on the repeatable battery for the assessment of neuropsychological status and their validation in an elderly sample. Arch Clin Neuropsychol.

