# Supplementary material for: Reduced microglia activity in patients with long-term immunosuppressive therapy after liver transplantation
Source: Eur J Nucl Med Mol Imaging. 2021 May 12;49(1):234–45. doi: 10.1007/s00259-021-05398-w (PMC8712291; doi:10.1007/s00259-021-05398-w)
Supplement: Supplementary file 1 — (DOCX 1.82 mb). [file 259_2021_5398_MOESM1_ESM.docx]

**Online Supplementary**

**REDUCED MICROGLIA ACTIVITY IN PATIENTS WITH LONG-TERM IMMUNOSUPPRESSIVE THERAPY AFTER LIVER TRANSPLANTATION**

**European Journal of Nuclear Medicine and Molecular Imaging**

Meike Dirks, Ralph Buchert, Ann-Katrin Wirries, Henning Pflugrad, Gerrit M. Grosse, Carlotta Petrusch, Christian Schütze, Florian Wilke, Martin Mamach, Linda Hamann, Laura B. N. Langer, Xiao-Qi Ding, Hannelore Barg-Hock, Jürgen Klempnauer, Christian H. Wetzel, Mario Lukacevic, Eike Janssen, Mariella Kessler, Frank M. Bengel, Lilli Geworski, Rainer Rupprecht, Tobias L. Ross, Georg Berding, Karin Weissenborn

**Corresponding author:**

Dr. med. Meike Dirks

Department of Neurology, Hannover Medical School

Carl-Neuberg-Str. 1, 30625 Hannover, Germany

Email: dirks.meike@mh-hannover.de

Phone: +49-511-532-3121 Fax: +49-511-532-3123

**Recruitment and eligibility criteria**

Patients with LT at least two years ago, age between 18 and 80 years, and stable immunosuppressive therapy regimen were included. Exclusion criteria were additional transplantation of other organs, liver re-transplantation (>three month after first LT), neurological or psychiatric diseases, regular intake of drugs affecting brain function, MRI contraindications, acute transplant-rejection or acute infection and decompensated heart-, liver- or kidney function.

**Polymorphism plot of ^18^F-GE-180 for the preparation of testing for an association between cognition data and TSPO expression**

Mean V_T_ in HAB control subjects was plotted versus the difference of mean V_T_ between HAB controls and MAB controls over all VOIs (Fig. S6). The x-intercept of the linear fit of the resulting scatter plot was used as estimate of V_ND_. The polymorphism plot assumes V_ND_ to be equal in all VOIs. Then, the specific distribution volume V_S_ of ^18^F-GE-180 for binding to TSPO was computed as V_S_ = V_T_ – V_ND_ for all HAB and MAB subjects and all VOIs. Next, assuming that (i) MAB subjects express 50% low affinity sites and 50% high affinity sites [14, 16, 22] and (ii) binding of ^18^F-GE-180 to low affinity sites can be neglected [20], the V_S_ was “corrected” for the genotype effect by multiplying V_S_ of MAB subjects by a factor 2 in all VOIs.

V_ND_ was estimated to 0.049 (ml/cm3), in good agreement with the mean V_T_ of 0.057 over all VOIs in the LAB control subject (Fig. S1).

**Supplementary table**

| Patients subgroup | **(1) CNI free** | | | | **(2) CNI low** | | | | **(3) CNI standard** | | | |
| --- | --- | --- | --- | --- | --- | --- | --- | --- | --- | --- | --- | --- |
| TSPO GT | All | LAB | MAB | HAB | All | LAB | MAB | HAB | All | LAB | MAB | HAB |
| n= | 3 | 1 | 1 | 1 | 9 | 0 | 2 | 7 | 10 | 2 | 5 | 3 |
| **Immunosuppression** | | | | | | | | | | | | |
| CsA |  |  |  |  |  |  |  |  | 3 |  | 2 | 1 |
| CsA + MPA |  |  |  |  | 1 |  | 1 |  | 1 |  | 1 |  |
| CsA + MPA + Prednisolone |  |  |  |  | 2 |  |  | 2 |  |  |  |  |
| Tac + Prednisolone |  |  |  |  | 1 |  |  | 1 | 1 |  | 1 |  |
| Tac + MPA |  |  |  |  | 4 |  | 1 | 3 | 4 | 2 |  | 2 |
| Tac + MPA + Prednisolone |  |  |  |  | 1 |  |  | 1 |  |  |  |  |
| Tac + Azathioprin + Prednisolone |  |  |  |  |  |  |  |  | 1 |  | 1 |  |
| Sirolimus + MPA | 1 | 1 |  |  |  |  |  |  |  |  |  |  |
| MPA + Prednisolon | 1 |  | 1 |  |  |  |  |  |  |  |  |  |
| Sirolimus + Prednisolon | 1 |  |  | 1 |  |  |  |  |  |  |  |  |
| **Aetiology of liver disease** | | | | | | | | | | | | |
| AIl diseases | 0 |  |  |  | 3 |  |  | 3 | 5 | 1 | 3 | 1 |
| HCV | 0 |  |  |  | 0 |  |  |  | 0 |  |  |  |
| HBV | 2 | 1 |  | 1 | 1 |  | 1 |  | 2 |  | 1 | 1 |
| Alcohol | 0 |  |  |  | 1 |  |  | 1 |  |  |  |  |
| ALF | 1 |  | 1 |  |  |  |  |  | 1 |  |  | 1 |
| Others | 0 |  |  |  | 4 |  | 1 | 3 | 2 | 1 | 1 |  |

**Table S1 TSPO-Genotype, immunosuppressive regimen and aetiology of liver disease for all patients included**

(CNI, Calcineurin inhibitors; GT, Genotype; LAB, low affinity binder, MAB; mixed affinity binder; HAB, high affinity binder, CsA, cyclosporine A; Tac, tacrolimus; MPA, mycophenolic acid; AI, Autoimmune; HCV, hepatitis C virus; HBV, hepatitis B virus; ALF, acute liver failure)

**Supplementary figures**

**
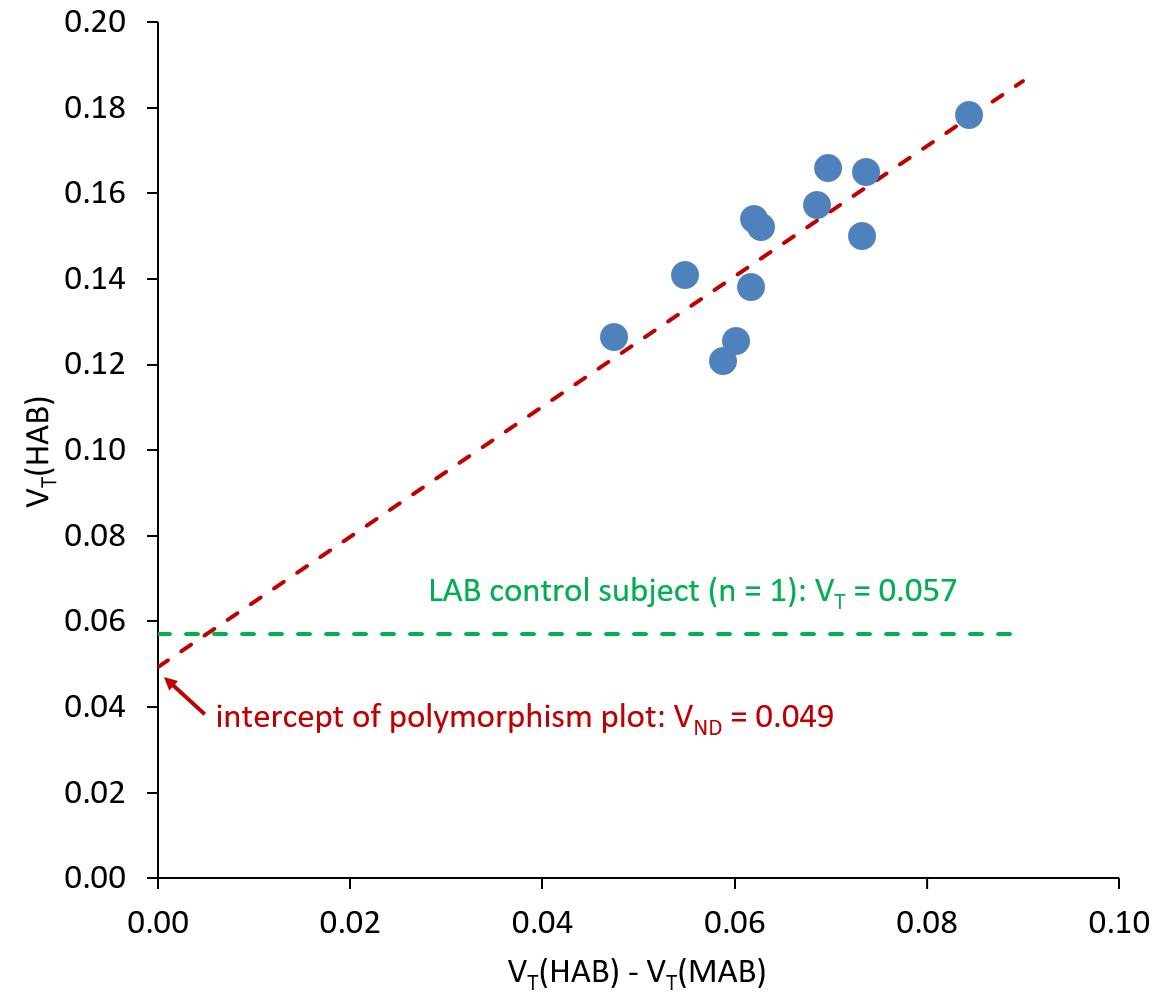
**

**Fig. S1 Polymorphism plot of ^18^F-GE-180**

The scatter plot shows the mean total distribution volume V_T_ in HAB control subjects versus the difference of mean V_T_ between HAB controls and MAB controls for all 12 VOIs (blue dots). The non-displaceable distribution volume V_ND_ was estimated to 0.049 by the intercept (arrow) of the regression line (dashed red line) fitted to the scatter plot. The dashed green line indicates the mean V_T_ (= 0.057) over all VOIs in the LAB control subject included in the study. This value also can be considered an estimate of V_ND,_ because specific binding of ^18^F-GE-180 most likely is negligible in LAB subjects. (V_ND_, non-displaceable distribution volume; V_T,_ total distribution volume; HAB, high affinity binder; MAB, mixed affinity binder; LAB, low affinity binder)

**
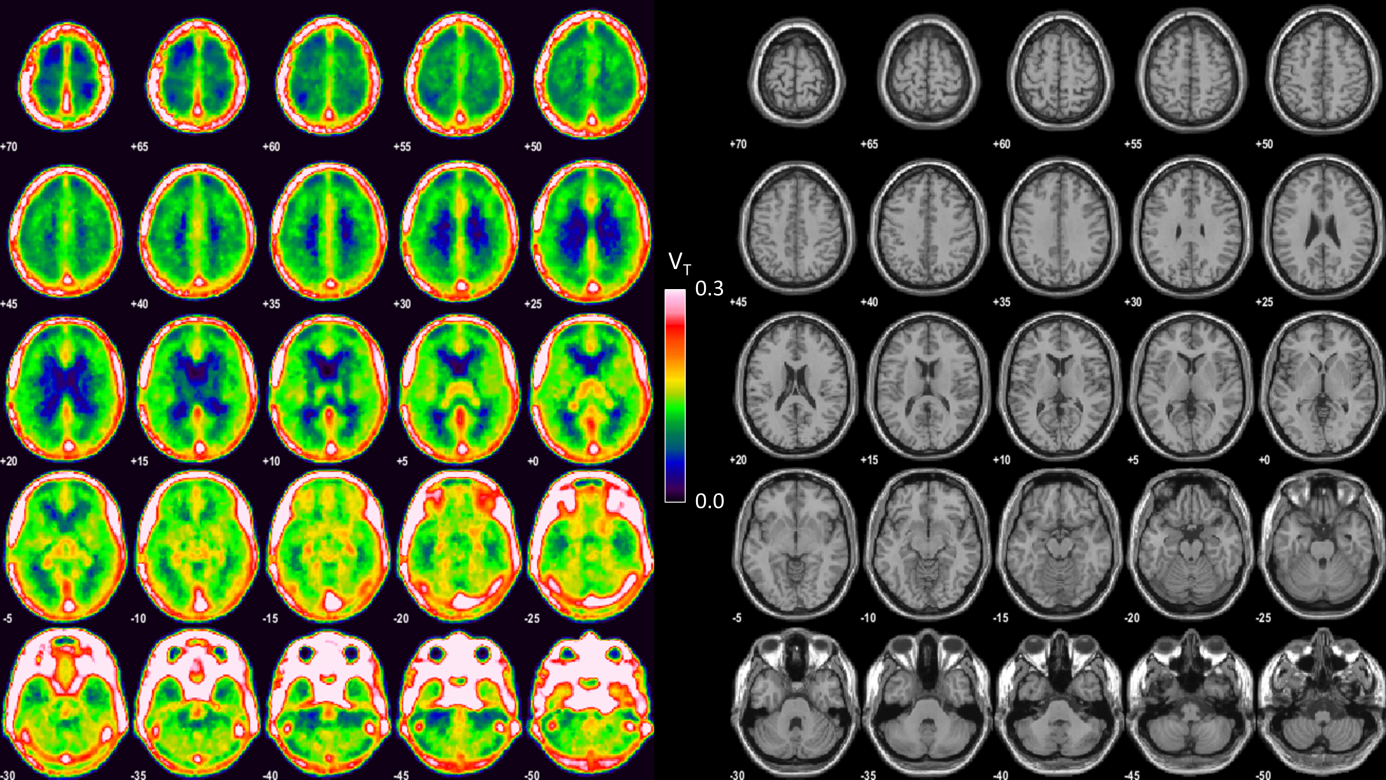
**

**Fig. S2** Mean V_T_ map in the five HAB controls (left). The single subject MRI template of SPM (right) is shown for better anatomical orientation.
